# Supplementary material for: WordCommentsAnalyzer: A windows software tool for qualitative research
Source: F1000Res. 2018 Sep 4;7:536. Originally published 2018 May 3. [Version 2] doi: 10.12688/f1000research.14819.2 (PMC6137411; doi:10.12688/f1000research.14819.2)
Supplement: Supplementary file 3 [file f1000research-7-17614-s0002.tgz › b2a79496-4ada-47c4-9682-a99ce867836d.zip!/Eight Iranian Health Journals/Yafteh _11.docx]

© Yafteh  . This work is licensed under CC BY 4.0 (https://creativecommons.org/licenses/by/4.0/).

Volume & Issue: دوره 17 - شماره 3

Title: ضرورت اجرای طرح ادغام افقی علوم پایه و رضایتمندی استادان و دانشجویان از اجرای طرح

http://yafte.lums.ac.ir/article-1-2064-fa.html

مقدمه: ادغام به ‌عنوان یک تحول در آموزش پزشکی در دانشگاههای جهان مطرح است و گذر از سیستم سنتی و تعریف جدید از آموزش پزشکی براساس نیازهای این دوره مطرح است. هدف این مطالعه ارزیابی نظرات و دیدگاههای اعضاء هیات علمی و دانشجویان پزشکی درگیر در پروژه ادغام علوم پایه می باشد. مواد و روش‌ها: این مطالعه یک مطالعه مقطعی بوده که در نیمه دوم سال 1393 در دانشکده پزشکی دانشگاه علوم پزشکی لرستان انجام شد. 10 استاد درگیر در اجرای طرح ادغام افقی و نیز 130 دانشجوی پزشکی مقطع علوم پایه در این مطالعه وارد شدند و با پرسشنامه هایی که روایی و پایایی آنها تایید شده بود، مورد بررسی قرار گرفتند. یافته‌ها: حدود 40٪ اساتید ضرورت طرح ادغام جهت تسهیل یادگیری دانشجویان و ضرورت طرح ادغام جهت درک بهتر مطالب و برقراری ارتباط بین دروس عملی و نظری را زیاد می دانستند و 70٪ اساتید ضرورت طرح ادغام جهت تطبیق این دروس با مسائل بالینی را زیاد می دانستند. بر اساس آزمون همبستگی پیرسون بین معدل کل و میزان رضایت از طرح ادغام دروس علوم پایه ارتباط معنادار دیده شد. دانشجویانی که معدل بیشتری داشتند میزان رضایتشان از طرح ادغام نیز بیشتر از سایر دانشجویان بود. بحث و نتیجه‌گیری: ادغام دروس علوم پایه جهت تطبیق دروس پزشکی در مقطع علوم پایه با مسائل بالینی مهم بوده ولی همچنان با مشکلاتی نظیر جلب رضایتمندی اساتید و دانشجویان در روش و ماهیت ادغام مواجه است که احتیاج به تحقیق بیشتری دارد.

Title: اثرات آنتی اکسیدانی و سیتوتوکسیسیتی عصاره آبی و الکلی میوه نسترن کوهی بر لاین سلولیU937

http://yafte.lums.ac.ir/article-1-2065-fa.html

مقدمه: گونه های فعال اکسیژن(ROS) می توانند برای سلول کشنده باشند.گیاهان خوراکی بعلت دارا بودن آنتی اکسیدان خطر بیماری و صدمات ناشی ازROS را پایین می آورد. هدف از این مطالعه تعیین اثر انتی اکسیدانی و سیتوتوکسیسیتی عصاره آبی و الکلی میوه نسترن کوهی بر لاین سلولیU937 است. مواد و روش‌ها: لاین سلولی U937 (سلول های لوکمیای انسانی) خریداری شده از انستیتو پاستور برای24 ساعت در انکوباتور قرار داده شد. سلول ها توسط آهن(50 میکرومولار) یا مس(50 میکرومولار) و H2O2(mM1%) دچار استرس اکسیداتیو شدند و به آن ها از هر کدام از مواد آنتی اکسیدان مورد نظر (عصاره ابی و الکلی) به طور جداگانه15 میکرو مول از غلظت های 100،50و25 میکرومولار اضافه شد (بجز نمونه های کنترل). سطوح گلوتاتیون توسط روش Tietze اندازه گیری شد. برای سنجش پراکسیداسیون لیپید از روش اندازه گیری مالون دی آلدیید و برای سنجش سیتوتوکسیتی از کیت LDH استفاده شد. تجزیه و تحلیل داده ها با آزمون های آنالیز واریانس و توکی انجام شد. یافته‌ها: میانگین و انحراف معیار غلظت مالون دی آلدیید در عصاره الکلی به ترتیب 6338/0 و0012/0میکرومول بر میلی گرم پروتیین و در عصاره آبی به ترتیب 6389/0 و 0017/0 در مقابل 6570/0 و 0023/0 برای کنترل بود(05/0 p< ) غلظت گلوتاتیون در غلظت های مختلف عصاره آبی و الکلی با کنترل تفاوت معنی داری نداشت. عصاره آبی و الکلی 25٪ نسبت به سایر غلظتها باعث کاهش بیشتر و معنی دار مالون دی آلدیید شد. عصاره الکلی 100 درصد دارای بیشترین (62/15%) و 25 درصد دارای کمترین (24/1%) سمیت بود. بحث و نتیجه‌گیری: عصاره الکلی و آبی میوه نسترن کوهی باعث کاهش پراکسیداسیون لیپید می شود و غلظت های بالای آن بیشترین سمیت را دارد.

Title: بررسی حذف دو-کلروفنل از محیط های آبی با استفاده از فرآیند الکترو/ پرسولفات

http://yafte.lums.ac.ir/article-1-2066-fa.html

مقدمه: دو-کلروفنل از جمله ترکیبات آلی آروماتیک می باشد که به مقدار زیاد از پساب صنایع نفت و پتروشیمی وارد محیط می‌شود که برای حذف آنها از روشهای شیمیایی مانند اکسیداسیون پیشرفته و غیره استفاده می کنند. از جمله روشهای مناسب اکسیداسیون پیشرفته، استفاده از رادیکال های سولفات ( SO4 ●- ) و پرسولفات (S2O82-) است که با حرارت، اشعه UV و فلزات واسطه نظیر یون های آهن فعال می گردد. هدف از این مطالعه کارایی پرسولفات فعال شده با یون های آهن حاصل از فرایند الکتروشیمیایی با الکترود آهن، برای حذف دو-کلروفنل است. مواد و روش‌ها: این مطالعه تجربی و در مقیاس آزمایشگاهی بود، که با استفاده از یک راکتور منقطع الکتروشیمیایی مجهز به 2 الکترود آهن متصل به منبع برق مستقیم انجام شد. مقادیر مختلف پارامترهای مؤثر بر فرآیند شاملpH، ولتاژ، غلظت اولیه پرسولفات و غلظت اولیه دو-کلروفنل بررسی شد. تعداد کل نمونه 234 عدد بود. بعد از جمع آوری داده ها، نتایج با استفاده از نرم افزارهای Excel و SPSS تجزیه و تحلیل شدند. یافته‌ها: نتایج نشان داد، pH اولیه محلول، غلظت اولیه پرسولفات و ولتاژ دارای تأثیر قابل توجهی در حذف دو-کلروفنل بودند. حداکثر میزان حذف در شرایط اسیدی و در pH برابر با 3 بدست آمد. همچنین با افزایش غلظت دو-کلروفنل، پرسولفات و ولتاژ، راندمان حذف افزایش یافت. به این ترتیب فرآیند تلفیقی الکترو/پرسولفات در مقادیر مناسب متغیرها، 94 /99 درصد دو-کلروفنل را حذف کرد. بحث و نتیجه‌گیری: استفاده از آنیون پرسولفات در راکتور الکتروشیمیایی مجهز به الکترود آهن، منجر به تولید رادیکال های سولفات شد، که کارایی بالایی درحذف دو-کلروفنل داشت. لذا فرآیند الکترو/ پرسولفات با الکترودهای آهن می تواند به عنوان گزینه ای مناسب در تصفیه خانه های فاضلاب های صنعتی مطرح گردد.

Title: بررسی اثر کوارستین بر شاخص‌های آنتی‌اکسیدانی سرم در آسیب ناشی از ایسکمی- ریپرفیوژن اندام تحتانی در موش صحرایی بالغ

http://yafte.lums.ac.ir/article-1-2067-fa.html

مقدمه: کوارستین یکی از اعضاء خانواده فلاونوئیدها می‌باشد که در سبزی‌ها، میوه‌ها، چای و در مکمل‌های غذایی یافت می‌شود. کوارستین خاصیت ضدالتهابی، آنتی باکتریال و آنتی‌اکسیدانی دارد. این مطالعه به بررسی اثر کوارستین بر روی شاخص‌های آنتی‌اکسیدانی سرم در آسیب ناشی از ایسکمی-ریپرفیوژن اندام تحتانی در موش بالغ می‌پردازد. مواد و روش‌ها: 72 موش نر بالغ نژاد ویستار با وزن 250-300 گرم استفاده شد. آن‌ها به 9 گروه هشت‌تایی تقسیم شدند. سپس شریان و ورید فمورال با استفاده از نخ سیلک 0/6 و فن Slip-Knot مسدود گردید. همه گروه‌ها به مدت 3 ساعت ایسکمی و ریپرفیوژن برای زمان‌های مختلف 3، 7، 14و28 روز اعمال شد. در نیمی از هر گروه تجربی مقدار mg/kg150 کوارستین به‌صورت درون صفاقی بلافاصله بعد از ایسکمی تزریق شد. طبق‌ زمان‌های مختلف برای هر گروه از موش‌ها خون‌گرفته شده و سپس سانتریفوژ و سرم آن‌ها برای ارزیابی سطح فعالیت آنزیم-های GPX،CAT، NO، POXو MDA تهیه گردید. یافته‌ها: مقایسه سطح سرمی فعالیت آنزیم‌های GPX،CAT، NO، POXو MDA در گروه دریافت‌کننده کوارستین نسبت با گروه کنترل نشان داد که کوارستین باعث کاهش سطح فعالیت سرمی NO و POXمی‌شود. بحث و نتیجه‌گیری: کوارستین با کاهش سطوح فعالیتNO می‌تواند آسیب ناشی از رادیکال‌های آزاد در فرآیند ایسکمی-ریپرفیوژن اندام تحتانی را بهبود بخشد.

Title: رفتارهای ارتقاء دهنده سلامت دانش آموزان دختر مقاطع متوسطه اول و دوم شهر پلدختر در سال 1392

http://yafte.lums.ac.ir/article-1-2068-fa.html

مقدمه: سلامت دختران نوجوان به دلایل متعدد فرهنگی و اجتماعی، ویژگی های بلوغ دختران و شرایط جسمی و روانی این دوران و از همه مهم تر نقش اساسی دختران در زمینه باروری و تأثیر آن بر زندگی خانواده ها در مقایسه با پسران از اهمیت بیشتری برخوردار است. پژوهش حاضر با هدف تعیین میزان رفتارهای ارتقا، دهنده سلامت دانش آموزان دختر شهر پلدختر صورت گرفت. مواد و روش‌ها: این مطالعه یک مطالعه مقطعی توصیفی- تحلیلی است. نمونه گیری از بین دانش آموزان به روش چند مرحله ای انجام گرفت. بدین صورت که هر کدام از مناطق به عنوان طبقه در نظر گرفته شد و مدارس دخترانه درون طبقات به عنوان خوشه انتخاب شدند. سپس به روش خوشه ای چند مرحله ای مدارس انتخاب و نمونه های مورد نظر به طور تصادفی انتخاب شدند. پرسشنامه مورد استفاده در پژوهش حاضر شامل پرسشنامه اطلاعات دموگرافیک و پرسشنامه استاندارد سنجش رفتارهای ارتقاء دهنده سلامت (HPLPII) بود. جهت تحلیل داده ها از آزمون t مستقل و آنالیز واریانس استفاده شده است. یافته‌ها: 421 پرسشنامه تکمیل گردید. میانگین نمره کلی رفتارهای ارتقاء دهنده سلامت 6/64 درصد کل نمره ممکن بود. میانگین سازه های مسئولیت پذیری سلامت، تغذیه، فعالیت فیزیکی و مدیریت استرس در دانش آموزان به ترتیب 7/68، 9/66، 2/58 و 8/56 درصد کل نمره ممکن بود. متغیرهای تحصیلات پدر و مادر، شغل مادر، بعد خانوار، نوع سرگرمی و نمایه توده بدنی با رفتارهای ارتقاء دهنده سلامت ارتباط معنی داری داشتند (001/0>p). بحث و نتیجه‌گیری: نتایج این پژوهش نشان داد که رفتارهای بهداشتی دانش آموزان دختر شهر پلدختر در حد متوسطی می باشد. دو سازه مدیریت استرس و فعالیت جسمانی نسبت به دو سازه دیگر وضعیت نامطلوب تری داشتند؛ لذا ضرورت توجه و مداخلات بیشتر را مبرهن می سازد.

Title: ارتباط الگوهای غذایی غالب و فاکتورهای اقتصادی- اجتماعی در میان کودکان روستایی سنین دبستان شهرستان بیجار در سال 1393

http://yafte.lums.ac.ir/article-1-2069-fa.html

مقدمه: شناسایی الگوهای غذایی غالب به روش تحلیل عاملی از طریق حصول یک تصویر کلی از رژیم غذایی افراد می‌تواند اطلاعات را در مورد وضعیت سلامتی کودکان در اختیار قرار دهد. هدف این مطالعه، تعیین الگوهای غذایی غالب و شناسایی عوامل اقتصادی-اجتماعی مؤثر بر آن‌ها در میان کودکان سنین دبستان روستایی شهرستان بیجار بود. مواد و روش‌ها: در این مطالعه مقطعی، 255 کودک روستایی سنین دبستان شهرستان بیجار به روش نمونه‌گیری تصادفی ساده انتخاب شدند. دریافت‌های غذایی در طول یک سال گذشته و اطلاعات اقتصادی- اجتماعی مورد ارزیابی قرار گرفت. الگوهای غذایی غالب به روش تحلیل عاملی تعیین گردید و ارتباط آن‌ها با وضعیت عوامل اقتصادی-اجتماعی مورد بررسی قرار گرفت. یافته‌ها: در این مطالعه سه الگوی غذایی غالب سنتی، امروزی و مخلوط شناسایی شدند. بعد از تعدیل برای سن، جنس، قومیت و انرژی دریافتی افراد تعدیل شده بود سن مادر(05/0-00/0=CI، 03/0=b) یک ارتباط مثبت و سن پدر(01/0--05/0-=CI، 03/0-=b)، شغل کارگری برای پدر(03/0--44/0-=CI، 24/0-=b) و تحصیلات بالاتر والدین(05/0--35/0-=CI، 20/0-=b) یک رابطه منفی با الگوی غذایی سنتی داشتند. همچنین تحصیلات والدین بالاتر (44/0-11/0=CI، 27/0=b) یک ارتباط مثبت و سن مادر(00/0-06/0-=CI، 03/0-=b) یک ارتباط منفی با الگوی غذایی مخلوط داشت. بحث و نتیجه‌گیری: بعضی از متغیرهای اقتصادی-اجتماعی می‌توانند بر روی الگوی های غذایی غالب در میان کودکان روستایی اثرگذار باشد که از آن جمله می‌توان به سن مادر، میزان تحصیلات والدین، شغل پدر و وضعیت اقتصادی اشاره نمود.

Title: اثرات آنتی‌اکسیدانی و تعدیل‌کنندگی ایمنی عصاره‌ برگ زیتون بر میزان سرمی سایتوکاین‌های مرتبط با سلول‌های Th17

http://yafte.lums.ac.ir/article-1-2070-fa.html

مقدمه: زیتون دارای اثر محافظتی اثبات شده در برابر بیماری‌های التهابی مزمن است؛ اما هنوز مشخص نیست که این خصوصیت زیتون ناشی از اثر آنتی‌اکسیدانی یا توانایی آن در تعدیل سیستم ایمنی است. هدف از این مطالعه، بررسی توانایی عصاره برگ زیتون بر سطح سرمی تعدادی از سایتوکاین‌های مرتبط با سلول‌های Th17 (به عنوان میانجی‌های مهم التهابی) و نیز اثر آنتی ‌اکسیدانی آن بر برخی از متغیرهای مربوطه در مغز موش صحرایی بود. مواد و روش‌ها: تعداد 40 رأس موش صحرایی نر به پنج گروه مساوی تقسیم شدند. به گروه اول شبه دارو، به گروه دوم ویتامین C با دوز mg/kg 10 و به سایر گروه‌ها عصاره برگ زیتون حاوی اولئوروپئین با دوزهای 5، 10 و 15 (mg/kg) به مدت ده روز به‌ صورت دهانی به وسیله گاواژ تجویز شد. در پایان دوره، خونگیری از قلب حیوانات انجام شد و میزان اینترلوکین‌های 17، 23 و TGF&beta; در سرم آن‌ها به روش الایزا اندازه‌گیری شد. فعالیت آنزیم‌های گلوتاتیون پراکسیداز (GPX)، سوپراکسید دیسموتاز (SOD)، کاتالاز (CAT) و غلظت مواد واکنش دهنده با اسید تیوباربیتوریک (به‌عنوان شاخص پراکسیداسیون لیپید یا TBARS) در نیم‌کره راست مغز ارزیابی شد. یافته‌ها: میزان TBARS در گروه کنترل نسبت به گروه‌های دیگر افزایش معنی‌داری را نشان داد (05/0< P). فعالیت آنزیم‌های GPX و SOD در گروهی که اولئوروپئین با دوزmg/kg 15 به آن‌ها تجویز شده بود نسبت به گروه کنترل و آن‌هایی که اولئوروپئین را با دوز کمتر دریافت کرده بودند افزایش آماری معنی‌داری را نشان داد (05/0< P). اگرچه در میزان IL-17 و IL-23 تفاوت آماری معنی‌داری بین گروه‌ها وجود نداشت، ولیTGF&beta; در گروه‌هایی که دوزهای مختلف اولئوروپئین را دریافت کرده بودند نسبت به گروه کنترل کاهش معنی‌داری را نشان داد (05/0< P). بحث و نتیجه‌گیری: عصاره برگ زیتون حاوی اولئوروپئین، دارای اثرات آنتی‌اکسیدانی معنی‌داری بر شاخص‌های اکسیداتیو در مغز موش صحرایی بود، ولی اثر قابل توجهی بر میزان سایتوکاین‌های مرتبط با سلول‌های Th17 (بجز (TGF&beta;نداشت؛ بنابراین اثر محافظتی آن در برابر بیماری‌های التهابی احتمالاً ناشی از خواص آنتی‌اکسیدانی آن است نه تعدیل سایتوکاین‌های مرتبط با سلول‌های Th17.

Title: مقایسه عوامل اتیولوژیک عفونت ادراری در بیماران بستری و سرپایی شهر گرگان

http://yafte.lums.ac.ir/article-1-2071-fa.html

مقدمه: عفونت دستگاه ادراری (UTI)یکی از مهمترین بیماریهای عفونی درمراجعین سرپایی و بیماران بستری می باشد. باکتری اشریشیاکلی شایع ترین ارگانیسم ایجاد کننده UTI بوده که به تنهایی مسئول بیش از80% عفونت ادراری است. هدف ازاین مطالعه،مقایسه عوامل اتیولوژیک عفونت ادراری در بیماران بستری و سرپایی شهر گرگان است. مواد و روش‌ها: این مطالعه توصیفی- مقطعی درسال91-90 بر روی 174 نمونه بیمار بستری و284 بیمار سرپایی انجام شد. برای هر فرد پرشسنامه ای شامل اطلاعات دموگرافیک پر شد. درآزمایشگاه، نمونه ها برروی محیط هایEMB، مکانکی و بلادآگار (شرکت لیوفیلکم)کشت شده و در oC37 برای 24ساعت انکوبه شدند. پس ازمشخص شدن گرم باکتریها، تست های اختصاصی و بیوشیمیایی بر روی نمونه ها صورت گرفت، نتایج با جدول استاندارد تشخیصی باکتری ها مقایسه شد. آنالیزآماری داده ها در برنامه SPSS 18 بررسی و 05/0&le; P به عنوان معنی دار در نظر گرفته شد. یافته‌ها: از 458 نمونه بررسی شده 38% در بیمارستان بستری بودند. 1/70% از 174 بیماربستری را زنان تشکیل می دادند. علائم بالینی نظیر، اورژانسی(6/23%) و تب (6/43%) در بیماران بستری بیشتر از بیماران سرپایی مشاهده شد. در بیماران بستری، اشریشیا کلی، فراوانترین میکروارگانیسم به میزان 3/48% جدا شد. سپس کلبسیلا با 3/10% ، انتروباکتر با 4/9%، پسودوموناس با 5/7%، انتروکوکوس با 1/1% ، پروویدنشیا با 1/1% ، سراشیا با 3/2% و ادواردسیلا با 2/0% فراوانترین باکتری ها در این گروه بودند. استافیلوکوکوس اورئوس فقط در بیمارن سرپایی جدا شد. فراوانی عوامل اتیولوژیک در بیماران بستری بطور معنی داری با افراد سرپایی تفاوت دارد. ارگانیسم های پروویدنشیا، سراشیا و ادواردسیلا فقط از گروه بیماران بستری در بیمارستان جدا شدند. بحث و نتیجه‌گیری: اشریشیا کلی در این مطالعه مشابه با سایر مطالعات صورت گرفته در نقاط مختلف ایران و جهان مهمترین پاتوژن در عفونت ادراری است فراوانی خانم ها درمقایسه با آقایان در ابتلا به عفونت های ادراری هم مانند اکثر مطالعات بسیار بیشتر بود که این ویژگی با وضعیت فیزیولوژیک دستگاه ادراری در خانم ها و آقایان ارتباط دارد.

Title: ارزیابی عملکرد بیمارستان شهید رحیمی‌خرم آباد براساس الگوی تعالی سازمانی EFQM، سال 1393

http://yafte.lums.ac.ir/article-1-2072-fa.html

مقدمه: در بخش بهداشت و درمان، همانند دیگر بخش‌ها، خودارزیابی امری اجتناب‌ ناپذیر است. محدودیت منابع این بخش، ضرورت پایش عملکرد، حیاتی بودن خدمات و مراقبت ‌های ارائه شده توسط بخش، اهمیت ویژه رعایت کیفیت در ارائه خدمات و ... شواهدی دال بر ضرورت خودارزیابی دقیق و صحیح عملکرد این بخش ‌هستند. از همین رو هدف اصلی این مطالعه، ارزیابی عملکرد بیمارستان شهید رحیمی‌ خرم آباد براساس الگوی تعالی سازمانی می باشد تا راهنمایی عملی برای بهبودخدمات این سازمان، در اختیار دست‌اندرکاران قرار دهد. مواد و روش‌ها: این مطالعه توصیفی مقطعی در بیمارستان شهید رحیمی‌ خرم آباد و در سال 1393 انجام گرفته است. ابزار گردآوری اطلاعات شامل پرسشنامه استاندارد خودارزیابی بر اساس الگوی تعالی سازمانی EFQM بوده که روایی و پایایی آن در مطالعات گذشته ارزیابی شده و ضریب آلفای کرونباخ آن 98 درصد برآورد شده است. یافته‌ها: درحوزه معیارهای توانمندسازی الگوی سرآمدی سازمانی، بعد رهبری دارای بهترین درصد امتیازی و بعد فرآیندها از کمترین امتیاز برخوردار بوده است که این بعد به همراه ابعاد کارکنان، منابع و شرکا، نیازمند بهبود می باشند. درحوزه معیارهای نتایج الگوی سرآمدی سازمانی، بعد نتایج مشتریان و نتایج کلیدی عملکرد دارای بهترین درصد امتیازی و بعد نتایج جامعه از کمترین امتیاز برخوردار بوده است که این بعد به همراه نتایج کارکنان، نیازمند بهبود می باشند. بحث و نتیجه‌گیری: یافته‌های این مطالعه در مجموع حاکی از وضعیت مناسب این بیمارستان از نظر معیارهای الگوی سرآمدی است. نتایج این مدل، عموماً برای تحلیل نظام مندسازمان به منظور رسیدن به نگرشی جامع از سازمان و چالشهای آن به کار گرفته می شود.

Title: بررسی رابطه بین عوامل سازمانی با سلامت روانی کارکنان سازمان‌های دولتی استان لرستان

http://yafte.lums.ac.ir/article-1-2073-fa.html

مقدمه: امروزه عوامل مختلفی مانند پیشرفت فناوری موجب گردیده که انسان کارها را سریع‌تر به انجام رساند؛ اما سرعت و دقتی که در انجام امور بر اثر این پیشرفت عاید بشر شده، موجب بروز برخی بیماری‌ها و ناراحتی‌های روانی و تضعیف روابط و ارزش‌های انسانی گردیده است. بنابراین هدف پژوهش حاضر بررسی رابطه بین عوامل سازمانی با سلامت روانی کارکنان سازمان‌های دولتی استان لرستان بود. مواد و روش‌ها: روش پژوهش توصیفی از نوع همبستگی بود. حجم نمونه این پژوهش را 379 نفر از کارکنان سازمان‌های دولتی استان لرستان تشکیل داد که با روش نمونه‌گیری طبقه‌ای نسبی انتخاب گردید. برای گردآوری داده ها از پرسشنامه‌های سلامت عمومی (28-GHQ)، سبک مدیریت باردنز و متزکاس، ابهام نقش و تعارض نقش جمشیدی‌نژاد، جو سازمانی سوسمان و دیپ و پرسشنامه محقق ساخته برای اندازه‌گیری نگرش کارکنان درباره عوامل سازمانی مرتبط با استرس استفاده شد. برای تحلیل داد ه‌ها از شاخص‌های توصیفی، ضریب همبستگی پیرسون، رگرسیون چندگانه به روش گام به گام، آزمون t برای گروه‌های مستقل و تحلیل واریانس چند متغیره استفاده شد. نرم‌افزار مورد استفاده نیز SPSS 19 بود. یافته‌ها: یافته‌ها نشان داد متغیرهای سازمانی معنی‌دار در پیش‌بینی سلامت روانی کارکنان سازمان‌های دولتی، شرایط کاری، جوسازمانی، سبک وظیفه‌مدار مدیر و ابهام نقش بودند. همچنین نتایج آزمون t‌ گروه‌های مستقل و تحلیل واریانس چند متغیره نشان داد که بین کل مقیاس سلامت روانی و مؤلفه‌های آن در کارکنان زن و مرد تفاوت معناداری وجود ندارد. بحث و نتیجه‌گیری: به طور کلی نتایج پژوهش حاضر حاکی از اهمیت نقش برخی از متغیرهای سازمانی در پیش‌بینی سلامت روانی کارکنان سازمان‌های دولتی استان لرستان بود.

Title: شناسایی الگوهای غذایی غالب در نوجوانان شهر کرج

http://yafte.lums.ac.ir/article-1-2074-fa.html

مقدمه: یکی از نگرانی‌ها در مورد سلامت عمومی عادات غذا خوردن نوجوانان است، زیرا ارتباط مستقیم بین رژیم غذایی ناکافی در این مرحله و بروز چاقی و دیگر بیماری‌های مزمن در بزرگسالی وجود دارد. شناسایی الگوهای غذایی می‌تواند به عنوان یک عامل پیش آگهی در ارتباط میان رژیم غذایی و خطر بیماری‌های مزمن استفاده شود. مواد و روش‌ها: مطالعه حاضر از نوع مطالعه مقطعی می‌باشد که بر روی 140 نوجوان سالم 19-13 ساله در مدارس راهنمایی و دبیرستان شهر کرج انجام شد. نمونه‌ها به روش نمونه گیری خوشه‌ای چند مرحله‌ای از 5 منطقه شهر کرج انتخاب شد. برای ارزیابی غذایی از پرسشنامه بسامد خوراک معتبر استفاده شد. اقلام غذایی در 25 گروه غذایی بر اساس شباهت طبقه بندی شدند. یافته‌ها: با روش تحلیل عاملی سه الگوی غذایی بدست آمد که 25/30 درصد از کل واریانس مصرف را توجیه می‌کردند. الگوی اول (شبه مدیترانه ای) که در آن مغزها، میوه‌ها و سبزیجات و ماهی، طیور، زیتون، شیرینی جات و ترشی جات مصرف بالایی داشتند. الگوی دوم (ناسالم)با مصرف بالای سس مایونز، غلات تصفیه شده، سیب زمینی آب پز، گوشت قرمز یا فراوری شده، لبنیات پرچرب و گوشت احشاء مشخص شد. الگوی سوم (سنتی) با مصرف بالای چربی‌های جامد، لبنیات کم چرب، حبوبات و غلات کامل تعیین شد. بحث و نتیجه‌گیری: الگوی شبه مدیترانه‌ای با 14/14 درصد بیشترین درصد واریانس را به خود تخصیص داده بود. با شناسایی این الگوهای می‌توان گام‌های آموزشی مناسبی در جهت ترویج تغذیه مناسب برداشت.

Title: فعالیت آنتی‌اکسیدانی و خاصیت ضد میکروبی دو نوع عسل حاصل از تغییر در جیره غذایی زنبور در مقایسه با دیگر عسل‌های تولیدی منطقه آبستان شهرستان خرم‌آباد

http://yafte.lums.ac.ir/article-1-2075-fa.html

مقدمه: عسل محصولی طبیعی از تراوشات گیاهان است که توسط زنبور عسل جمع آوری و پس از ایجاد تغییراتی، در کندو ذخیره می‌شود. عسل غنی از آنتی‌اکسیدان‌های مختلف از جمله کاتالاز، اسید آسکوربیک، فلاونوییدها و آلکالوییدها می‌باشد. فعالیت ضد میکروبی عسل نیز یکی از جنبه های مهم آن است. وارد نمودن برخی مواد به جیره غذایی زنبور عسل نیز می‌تواند بر ارزش غذایی عسل تولیدی بیافزاید. لذا در این مطالعه فعالیت آنتی اکسیدانتی و خاصیت ضد میکروبی دو نوع عسل حاصل از تغییر در جیره غذایی زنبور بررسی و با سایر عسلهای تولیدی منطقه مقایسه می‌گردد. مواد و روش‌ها: سه نوع تغذیه مختلف شامل آب سیب، عصاره ریشه و گلهای‌ شیرین بیان و گل‌های منطقه بدون تأکید بر گیاه خاصی برای زنبورها در نظر گرفته شد. فعالیت ضد میکروبی سه نوع عسل تولیدی بر روی باکتری‌های استاندارد و مخمر کاندیدا البیکنس با استفاده از روش انتشار در آگار در مقایسه با چند آنتی بیوتیک رایج اندازه گیری شد. برای اندازه گیری مقدار ترکیبات آنتی اکسیدانی، آزمون فنل تام و برای ارزیابی خواص آنتی اکسیدانی از آزمون‌های دی فنیل پیکریل هیدرازیل، فعالیت آنتی اکسیدانی معادل ترولکس و فعالیت آنتی اکسیدانی آهن احیا شده، استفاده گردید. یافته‌ها: عسل شیرین بیان بیشترین فعالیت ضد میکروبی را علیه باکتری‌ها و مخمر را در این بررسی بخصوص علیه سودوموناس آئروجینوزا از خود نشان داد. فعالیت آنتی اکسیدانی عسل‌های مورد آزمایش دارای اختلاف معنی‌دار از نظر مقدار فنل تام بودند و عسل سیب در سه روش ارزیابی، خواص آنتی اکسیدانی بیشتری از خود نشان داد. بحث و نتیجه‌گیری: این با توجه به نتایج این تحقیق، تغذیه زنبورها با گیاهان دارای مواد مؤثره باعث ایجاد تحول در بخش تولید عسل دارویی و ارتقای سلامت جامعه می‌گردد.
